# Utility of FDG PET/CT for assessment of lung nodules identified during low dose computed tomography screening

**DOI:** 10.1186/s12880-020-00469-0

**Published:** 2020-06-22

**Authors:** Sarah Hadique, Pranav Jain, Yousaf Hadi, Aneeqah Baig, John E. Parker

**Affiliations:** 1 Section of Pulmonary, Critical Care & Sleep Medicine, West Virginia University, 1 medical center drive, HSC-N 9166, Morgantown, WV 26506 USA; 2 Fourth year Medical student, West Virginia University, Morgantown, WV USA; 3 Department of Internal Medicine, West Virginia University, Morgantown, WV USA

**Keywords:** LDCT, Lung cancer, FDG PET/CT, Pulmonary nodule

## Abstract

**Background:**

Many clinical guidelines recommend FDG PET/CT for the evaluation of pulmonary nodules ≥8 mm detected during low dose computed tomography (LDCT) lung cancer screening. However, its added value in this setting requires confirmation. We evaluated the clinical utility of FDG PET/CT, including incidental findings, during the evaluation of lung nodules detected on LDCT screening.

**Methods:**

A retrospective cohort study was performed among 75 patients who completed FDG PET/CT between January 2010 and December 2017, after lung nodules > 8 mm had been detected on LDCT lung cancer screening. We report demographic variables, characteristics of the initial nodules on LDCT and FDG PET/CT, incidental findings on FDG PET/CT, as well as further work up performed and the influence of FDG PET/CT findings on management.

**Results:**

Nodules were reported to be benign on FDG PET/CT in 38/75 (50.6%) patients. Physicians chose either radiological follow-up or no further work up in all 38. FDG PET/CT was indeterminate or suggested malignancy in 37 (49.3%) patients. Biopsy was performed in 32 (86%) of these patients. Incidental findings on FDG PET/CT were reported in 37/75 (49%) patients. Further work-up of incidental findings was performed in 21/75 (28%) of patients.

**Conclusions:**

In this study, for majority of individuals with lung nodules identified during LDCT lung cancer screening, FDG PET/CT results were able to guide physicians in choosing between routine follow up or invasive biopsies. Conversely, 28% of these patients required additional investigations to address incidental findings.

## Background

Screening with low dose chest computed tomography (LDCT) has been shown to reduce lung-cancer-related mortality by 20% [1]. Currently, annual screening for lung cancer with LDCT is recommended in adults aged 55 to 80 years who have a 30 pack-year smoking history and currently smoke or have quit within the past 15 years [2–4]. However, 96% of the nodules detected on LDCT are non-malignant and any further evaluation of these nodules can add to the cost and procedure-related complications [5].

For lesions ≥8 mm the average risk of malignancy is 3% [6, 7]. To date, there is no universally accepted clinical pathway for evaluation of nodules detected on LDCT. A repeat chest computed tomography (CT) after 3 month, [^18^F] fluorodeoxyglucose positron emission tomography – computed tomography (FDG PET/CT), or tissue sampling are all acceptable to investigate these lesions [7–9]. FDG PET/CT has been used for nearly two decades for diagnosis and staging of lung cancer with sensitivity and specificity of about 90 and 75%, respectively [10, 11]. However, there is limited information on clinical application of FDG PET/CT to assessment of nodules discovered on LDCT screening [7, 12].

Due to this uncertainty, utilization of FDG PET/CT in these patients is highly variable. For example, in a recent Canadian study, of 139 lung nodules detected on LDCT, FDG PET/CT was not performed in any patient [13]. In other studies, FDG PET/CT was performed in 3.1 to 6.5% of subjects with lung nodules detected on LDCT [14, 15].

For LDCT detected nodules, there is limited data on the impact of FDG PET/CT on clinical decisions [16]. Arguably, a routine FDG PET/CT for every lung nodule might add significantly to the cost of care. A further concern is that FDG PET/CT may detect additional findings of little clinical significance. Nevertheless, addressing these findings may further increase the cost of care.

To address these issues, we performed a retrospective cohort study to investigate how FDG PET/CT results affected management of nodules detected on LDCT, as well as the detection and subsequent workup of incidental findings.

## Methods

### Study population

A structured medical record review was performed for patients who underwent a FDG PET/CT for further assessment of lung nodules detected during LDCT screening for lung cancer in the Department of Radiology at West Virginia University (WVU) between Jan 2010 and Dec 2017. Patients were identified using relevant Current Procedural Terminology (CPT) codes, and were excluded if biopsies or additional imaging studies were completed before the FDG PET/CT. All LDCT and FDG PET/CT were performed at WVU and read by the two staff radiologists. Standard patient preparation protocols and procedures were followed. Standardized uptake value (SUV) cutoff of 2.5 was used for nodules > 8 mm and delayed time images were also acquired at 2 h. The final report was generated in relation to other clinical (age, smoking history) and radiologic (spiculation) factors determining the likelihood of malignancy. The study protocol was approved by the institutional review board of WVU (Protocol Number: 1805111017).

### Data collection

Data were extracted from the institutional electronic health record system (EPIC) by two independent investigators. Demographic variables (age, gender), co-morbid conditions, and the number, location, size, and morphology of the lung nodules on initial LDCT were recorded. FDG PET/CT report was reviewed to determine whether the nodule was reported as benign, indeterminate or malignant. A careful review of each medical record was performed to determine the course of action taken after the ordering physician received the FDG PET/CT report. Whether FDG PET/CT findings affected the decision to pursue serial radiological follow up, perform biopsy, or surgery was recorded through clinician’s progress notes. We also determined if FDG PET/CT findings allowed clinicians to choose an alternative and more approachable site of biopsy. A final determination of malignancy was made on histology except for two patients who declined biopsy where FDG PET/CT was consistent with widely metastatic disease. Lung nodules were deemed benign if interpreted as such on FDG PET/CT and remained unchanged on serial imaging for a period of 2 years. Additional findings on FDG PET/CT were recorded if they were not present on LDCT. Subsequent biochemical tests, consultations, diagnostic imaging or biopsy etc. were documented if they addressed the additional findings.

### Statistical analysis

Statistical analysis was performed using the R statistical package. Mean and standard deviation were calculated for continuous variables and proportions were calculated for categorical variable. 95% confidence intervals were calculated for relevant variables. Continuous variables were compared using two-sided student t- test and proportions were compared using Chi-square test. A *p* value of < 0.05 was considered statistically significant.

#### Patient and public involvement

This was a retrospective chart review using the institutional electronic health record system (EPIC), without patient or public involvement.

## Results

**Fig. 1:**
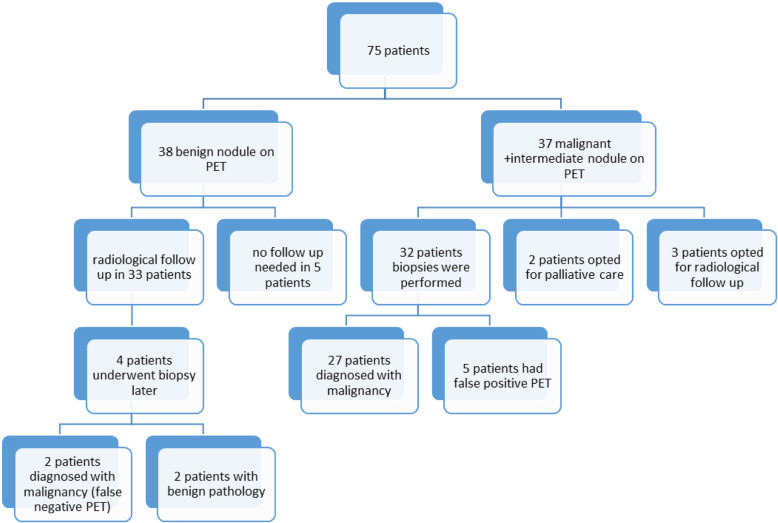
FDG PET/CT Positron Emission Tomography-Computed Tomography Results and Clinical Outcome of 75 Study Patients

A total of 75 patients fulfilled criteria for inclusion (Fig. 1). Mean age was 64.7 ± 7.8 years and 39 (52%) patients were females. Right upper lobe was the most common location of the nodules (23/75, 30.6, 95% CI 20–41%). Most lung nodules were solid (59/75, 78.6, 95% CI 69–88%) and ≥ 10 mm in size (60/75, 80, 95% CI 71–89%) (Table 1).

**Table 1:** Demographic Data (*n* = 75)

Age (years)	64.7 ± 7.8
**Gender (n, %)**	
Male	36 (48%)
Female	39 (52%)
**Comorbid conditions (n, %)**	
COPD	55 (73.3%)
Hypertension	44 (58.6%)
Hyperlipidemia	32 (42.6%)
Depression	17 (22.7%)
Diabetes Mellitus	15 (20%)
History of malignancy	7 (9.3%)
Single nodule (n, %)	35 (46.6%)
Multiple nodule (n, %)	40 (53.3%)
Solid nodule (n, %)	59 (78.6%)
**Nodule size (n, %)**	
Size < 8 mm	4 (5.3%)
Size 8–9 mm	11 (14.6%)
Size 10–19 mm	38 (50.6%)
Size ≥20 mm	22 (29%)

Nodules were reported to be benign on FDG PET/CT in 38 (50.6, 95% CI 39–62%) and malignant or indeterminate in 37 (49.3, 95% CI 38–60%) of patients. Hypermetabolic hilar or mediastinal lymph nodes were detected in 12 (16, 95% CI 8–24%) of patients. Average size of benign nodule was 13.7 ± 10.5 mm, whereas the average size of malignant or indeterminate nodules was 20.8 ± 9.9 mm (*p* < 0.005) (Table 2).

**Table 2:** FDG PET/CT Positron Emission Tomography – Computed Tomography Findings

Characterization on FDG PET/CT	N (%)
Benign	38 (50.6%)
Malignant	31 (41.3%)
Intermediate	6 (8%)
Hilar and Mediastinal lymphadenopathy	12 (16%)
Average size of benign nodule (size± SD)	13.7 (±10.5) mm
Average size of malignant nodule (size ± SD)	20.8 (±9.9) mm
**Location of dominant/ largest nodule**	
Right upper lobe	23 (30.6%)
Right middle lobe	6 (8%)
Right lower lobe	18 (24%)
Left upper lobe	18 (24%)
Left lower lobe	9 (12%)
No dominant nodule	1 (1.3%)

Out of 38 patients with benign report on FDG PET/CT, physicians decided to pursue radiological follow up in 33 (87, 95% CI 72–96%) of these patients. No further work up or follow up imaging was performed in 5 patients (13, 95% CI 4–28%) after resolution or decrease in size of nodules between LDCT and FDG PET/CT. Four patients with benign report on PET/CT later underwent biopsies (wedge resections and CT guided biopsies in 2 patients each), and pathology revealed fibrotic changes and carcinoid tumors in 2 patients each. No diagnosis of malignancy was made during a 2-year follow up in remaining 36 patients initially thought to have benign nodules on FDG PET/CT.

Of the 37 patients with indeterminate or malignant reports, clinicians chose radiological follow up in three patients with indeterminate nodule. Nodules remained stable in size in all three patients over 2 years. Two patients were found to have widespread metastatic disease and declined biopsies or any other further work up and opted for palliation directly after discussion of FDG PET/CT findings. The remaining 32/37 patients (86, 95% CI 75–97%) with malignant (*n* = 29) or indeterminate (*n* = 3) FDG PET/CT reports underwent biopsy procedure. All percutaneous biopsies were performed under CT guidance. Ultrasound guidance was not used in any biopsy procedure. FDG PET/CT finding was proven false positives in 5 of 32 patients who had biopsy procedure. In these patients, nodules were suggested to be to be malignant on FDG PET/CT, but biopsy revealed granulomatous diseases in 3 patients, amyloidosis in 1 patient and hamartoma in 1 patient. Biopsy confirmed malignancy in remaining 27 patients in which two were from extrathoracic site. FDG PET/CT assisted with not only diagnosis but staging of lung cancer. Adenocarcinoma was the most common neoplasm, identified in 15 patients. The rest were squamous cell, small cell, neuroendocrine and undifferentiated carcinomas. Overall, in the study cohort, 31 of 75 (41.3, 95% CI 30–52%) were diagnosed to have malignancy.

Using biopsy or two-year stability to establish final diagnosis, the overall, the sensitivity, specificity, positive predictive value and negative predictive value of FDG PET/CT in our study were 94, 82, 78 and 95% respectively.

A total of 55 extra-thoracic incidental and clinically unsuspected findings were detected in 37 of all study patients (49.3, 95% CI 38–61%). Further work up to address these findings was pursued in 21 (28%) of patients. These included additional imaging to in 7 (9%) invasive biopsy in 3 patients (4%), consultation from other services in 5 (7%) and biochemical testing in 6 (8%) patients. Biopsy showed benign pathology in one, and hematological malignancy unrelated to lung nodule in two patients. One patient with a complex ovarian mass with suspected metastasis opted for palliation therapy.

## Discussion

There is limited information on how nodules detected during screening LDCT are managed in clinical practice. We report diagnostic interventions and two-year health outcomes among 75 patients who underwent FDG PET/CT after one or more lung nodules were identified during LDCT cancer screening. (1) The main findings are: (1) Physicians chose noninvasive radiological follow up for all lung nodules interpreted as benign on FDG PET/CT, (2) In contrast, biopsy was performed in 86% of patients when FDG PET/CT suggested malignant or indeterminate nodules, (3) In this setting, FDG PET/CT performed well, with a sensitivity and specificity, PPV and NPV of 94, and 82%, 78 and 95% respectively, (4) Incidental findings on FDG PET/CT were very common (49%), and these triggered further workup in a significant proportion of patients.

Although several risk prediction models have been devised recently, they may not offer a significant improvement in distinguishing benign versus malignant nodule over clinical judgment alone [17]. Our results show that physicians in practice are able to effectively select very different approaches to lung nodules on the basis of FDG PET/CT results.

FDG PET/CT suggested benign etiology in 51% of our study patient. Invasive biopsy was not pursued any patient with a benign FDG PET/CT report. Malignancy was subsequently detected in two patients and in both cases the underlying pathology was peripheral carcinoid. This is not unexpected since carcinoid tumor is a well-established cause of false negative FDG PET/CT performed for solid lung nodules [18]. Among patients with a negative FDG PET/CT, 87% of the were followed with serial radiological examinations to provide additional reassurance regarding benignity of nodules. In essence, a high NPV of 95% in our study was sufficient for clinicians to withhold immediate invasive procedures but not enough to conclude benignity without further radiological follow up. Lack of growth in lung nodules on follow up imaging provided a further reassurance to the clinicians, as previously reported [19].

In a striking contrast, physicians chose to pursue immediate biopsy in 86% of patients with FDG PET/CT reported as malignant or indeterminate. Only 3 (8%) of patients with indeterminate FDG PET/CT report were followed with serial radiological examination. The positive predictive value of a malignant or indeterminate report on FDG PET/CT was 78% in our patients, while the negative predictive value was 95%. Thus, a positive FDG PET/CT clearly helped clinicians to choose biopsy over radiological follow up in majority of patients.

In 3 study patients, FDG PET/CT provided important information on the widespread nature of malignancy, which was helpful in choosing hospice and palliative care. Further diagnostic or therapeutic measures directed at cancer were avoided in these patients. Clearly, in these patients.

Incidental findings were very common, reported in 49% of patients undergoing FDG PET/CT. Further work up to address these findings was pursued in 28% of patients. Notably, further work up was largely non-invasive such as further imaging and consultations. Invasive biopsies of extra-thoracic sites on the basis of FDG PET/CT were pursued in 3 patients, and 2/3 confirmed cancers. Therefore, our data show that physicians were selective in addressing incidental findings and invasive testing was rarely pursued.

While FDG PET/CT may seem to have increased the direct cost of care, our data shows that It also allowed physicians to avoid invasive testing in more than one half of our study patients. Arguably, in the absence of reassurance from negative FDG PET/CT results, a biopsy would have been pursued in a significant proportion of these patients. The overall cost of care increases by several folds when biopsy is performed for assessment of lung nodules. A previous study on a Medicare subsample has shown that median diagnostic cost per patient for those with biopsy versus without biopsy was approximately 28 times higher [20]. A recent study has also reported a complication rate can be as high as 23.8% from invasive testing for lung nodules in Medicare patient [21]. Thus, rather than increasing the cost of care, selective use FDG PET/CT may have actually resulted in a significant cost saving by avoiding unnecessary biopsy procedures and attendant complications in these patients. Our findings help to justify a formal cost-effectiveness analysis on use of FDG PET/CT in assessment of LDCT detected lung nodules.

These results are limited by the retrospective design, single center, and modest sample size. Also, the information on additional work up addressing incidental findings was limited to what was performed or documented at our institution only. We cannot exclude the possibility of additional testing done elsewhere by referring physicians. Further, we are unable to determine the reasons that contributed to the decision by physicians to obtain FDG PET/CT after detection of lung nodules on LDCT. Size and radiological appearance of nodule, and suggestion by the radiologist may have played a role. Interestingly, 41% prevalence of malignancy in our study is remarkably similar to 38% prevalence of malignancy in another study on patients undergoing FDG PET/CT after a LDCT [22]. There is need for prospective data to identify patients who are most suited to undergo FDG PET/CT after a lung nodule is identified on LDCT rather than serial CT scans or an upfront invasive biopsy. In this context, it is also important to limit the cumulative radiation exposure to the patients found to have lung nodules on LDCT. Studies are needed to better define the role of percutaneous ultrasound guided biopsies in LDCT detected nodules located in sub-pleural location. We believe that radiologists interpreting LDCT images can play a pivotal role in guiding clinicians regarding the suitability to undergo ultrasound guided procedure if biopsy is a consideration. Importance of a direct discussion between the clinician and radiology consultant in such decision making cannot be overstated.

Additionally, many patients at our hospital reside in a region with potential for environmental exposures to histoplasma [23]. Granulomatous lung lesions in the screened population might potentially affect the operating characteristics of the PET/CT test for cancer, although the effect has not been noted in recent screening programs [24].

Despite these limitations, our data clearly shows that FDG PET/CT performed in lung nodules ≥8 mm in size detected on screening LDCT is very helpful in making important clinical decisions. We also show that despite common reporting of incidental findings, physicians in this study showed considerable restraint in pursuing invasive work up to address these incidental findings. Future studies are urgently needed to identify factors that increase likelihood of malignancy in a LDCT detected nodule. Limiting FDG PET/CT to that subset is likely to improve cost-effectiveness in managing LDCT detected nodules.

## Conclusion

FDG PET/CT was shown to be a useful test in further assessment of lung nodules detected on LDCT. Our findings demonstrate that FDG PET/CT can furnish increased confidence for clinicians when selecting diagnostic alternatives for patients with lung nodules detected during LDCT lung cancer screening.

## Data Availability

The datasets used and/or analyzed during the current study are available from the corresponding author on reasonable request.

## Abbreviations

ACCP: American college of chest physicians
CPT: Current procedural terminology
CT: Computed tomography
EPIC: Electronic health record system
LDCT: Low dose chest computed tomography
NCCN: National Cancer Center Network
PET: Positron emission tomography
FDG: [^18^F] Fluorodeoxyglucose
